# Assorted Methods for Decontamination of Aflatoxin M1 in Milk Using Microbial Adsorbents

**DOI:** 10.3390/toxins11060304

**Published:** 2019-05-29

**Authors:** Jean Claude Assaf, Sahar Nahle, Ali Chokr, Nicolas Louka, Ali Atoui, André El Khoury

**Affiliations:** 1 Centre d’Analyses et de Recherche (CAR), Unité de Recherche Technologies et Valorisation agro-Alimentaire (UR-TVA), Faculté des Sciences, Université Saint-Joseph de Beyrouth, Campus des sciences et technologies, Mar Roukos, Matn 1104-2020, Lebanon ; sahar.nahle@hotmail.com (S.N.); nicolas.louka@usj.edu.lb (N.L.); andre.khoury@usj.edu.lb (A.E.K.); 2 Research Laboratory of Microbiology, Department of Life and Earth Sciences, Faculty of Sciences I, Lebanese University, Hadat Campus, Beirut P.O Box 5, Lebanon; alichokr@hotmail.com (A.C.); aatoui@ul.edu.lb (A.A.); 3 Platform of Research and Analysis in Environmental Sciences (PRASE), Doctoral School of Sciences and Technologies, Lebanese University, Hadat Campus, Beirut P.O. Box 6573/14, Lebanon

**Keywords:** decontamination, mycotoxins, Aflatoxin M1, milk, binding, stability

## Abstract

Aflatoxins (AF) are carcinogenic metabolites produced by different species of *Aspergillus* which readily colonize crops. AFM1 is secreted in the milk of lactating mammals through the ingestion of feedstuffs contaminated by aflatoxin B1 (AFB1). Therefore, its presence in milk, even in small amounts, presents a real concern for dairy industries and consumers of dairy products. Different strategies can lead to the reduction of AFM1 contamination levels in milk. They include adopting good agricultural practices, decreasing the AFB1 contamination of animal feeds, or using diverse types of adsorbent materials. One of the most effective types of adsorbents used for AFM1 decontamination are those of microbial origin. This review discusses current issues about AFM1 decontamination methods. These methods are based on the use of different bio-adsorbent agents such as bacteria and yeasts to complex AFM1 in milk. Moreover, this review answers some of the raised concerns about the binding stability of the formed AFM1-microbial complex. Thus, the efficiency of the decontamination methods was addressed, and plausible experimental variants were discussed.

## 1. Introduction

Aflatoxins (AF) are secondary metabolites produced by several *Aspergillus* species, mainly by *Aspergillus flavus* and *A. parasiticus* [1,2,3,4]. The AF contamination of food and feed after mold colonization may occur at any stage extending from pre-harvest to consumption [5,6]. Thus, this can cause the direct or indirect contamination of different food commodities including cereals, corn, rice, and peanuts. Humid and warm environments are suitable for mold growth and AF production [7,8]. The group of AF includes more than 20 known metabolites; the most important are the naturally occurring ones such as B1, B2, G1, and G2 [9,10,11]. The toxicity of AF varies, but AFB1 remains the most toxic for humans and animals [12,13,14]. Briefly, after ingestion and absorption by an animal’s gastrointestinal tract, AFB1 is then transformed in the liver into AFM1 and aflatoxin M2 (AFM2) [15,16,17,18]. It is noteworthy to mention that milk and its derivatives are widely consumed not only by adults but, more importantly, by infants [19]. Interestingly, Williams et al. reported that more than 4.5 billion people worldwide are at risk of exposure to foodstuffs contaminated with different levels of AF [20]. Upon investigation of its toxicity, the International Agency for Research on Cancer (IARC) has classified AFM1 as a group 1 human carcinogen [21,22,23]. The secretion of AFM1 in milk varies widely according to different factors including animal species, season and milking time, level of AFB1 intake, and volume of milk produced by the mammal in question [24,25,26]. Once in milk, AFM1 is not degraded and can resist different industrial treatments including milk sterilization or pasteurization, in addition to any other heat treatments [27,28,29,30,31]. For this reason, AFM1 contamination remains a serious problem, not only in produced milk but also in all its derived products including cheese, yoghurt, cream, and powdered milk [32,33]. Due to AF’s harmful effects, several countries and international organizations have strictly regulated AF levels in feed and food [34,35]. Thus, the highest acceptable level of AFM1 in milk ranges between 0.05 μg/kg and 0.5 μg/kg, as established, respectively, by the European Union (EU) and the Food and Drug Administration (FDA) [36,37]. Therefore, the adopted AFM1 limit in milk (0.5 μg/kg) settled up in the USA, Brazil, Japan, and India is less restrictive compared to other countries such as France, Germany, Belgium, Australia, and Turkey (0.05 μg/kg) [38]. Importantly, the stricter the regulatory limits, the more food commodities are wasted, which results in a higher economic loss [39,40]. Accordingly, these regulations depend on several factors including the economic development level of each country, limits of consumption, and risk of exposure to AFM1 [41]. Moreover, the trade of any AF-contaminated products was also prohibited [40]. The implementation of Good Agricultural Practices (GAP) remains the best way to limit AF contamination in food and feedstuff but cannot guarantee their absolute prevention [42,43,44]. In addition, innovative technologies to cut pre- and post-harvest exposure to AF are strongly recommended. Some of these technologies include ozone fumigation [45], irradiation biological [46,47], and chemical agents [40,48,49]. Highly promising techniques such as using the biofilms of probiotic bacteria [50], chitin, and treated crustacean shells [51] are under rigorous investigation. Furthermore, the use of different biotransforming agents such as microorganisms and their purified enzymatic products can lead to the catabolization, cleaving, or transformation of the AF molecule to less or non-toxic metabolites [52]. Similarly, several clay materials including bentonite, hydrated sodium calcium aluminosilicate (HSCAS), zeolite, and activated charcoal have shown varying abilities to reduce AF in contaminated feedstuff [52]. Up until now, the most studied methods to mitigate AF contamination are mainly based on using biological adsorbents such as bacteria and yeasts [52,53,54].

This review aims to critically discuss different methods for AFM1 decontamination by microbial adsorption. Therefore, various treatments used for AFM1 decontamination by yeasts or bacteria will be broadly scrutinized, and some experimental variants will be highlighted in order to help researchers in improving the commonly used methods.

## 2. Toxic Effects of AFM1

Amongst all mycotoxins, AF present a high risk on the human health due to consumption of foods, including milk and dairy products, contaminated with their derivatives such as AFM1 [55,56]. Hence, the potential existence of AFM1 in milk, even in minor quantities, remains a worldwide alarming issue due to the consumption of wide range of contaminated dairy products [38]. Accordingly, the International Agency for Research on Cancer (IARC), following investigations on its toxicity, shifted AFM1 classification from group 2B to group 1 human carcinogens [22,57].

Briefly, AFB1 is initially absorbed by the gastrointestinal tract before being metabolized in the liver [58,59]. Within 15 min after ingestion, AFM1 could be detected in the blood of the lactating animal before being secreted in its milk and urine [15,58,60]. The biotransformation of AFB1 in the animal liver is carried out by cytochrome P450 enzymes, thus metabolizing AFB1 into hydroxylated AFM1 and AFB1 reactive epoxides [61,62]. While AFM1 is less toxic than AFB1, it is still highly harmful for humans and animal species [16,63]. As the major organ targeted by AFM1 is the liver, it is considered a hepatotoxic metabolite [64,65]. In addition, other damaging effects including immunity suppression, reduced milk production, and lower oxygen supply to body tissues may be caused by AFM1 [66,67,68]. The toxicity of AF, including AFM1, and its impact on its host is sex, age, species, and nutritional-behavior dependent [20,69]. It is important to highlight that breast-feeding is always encouraged for infants due to its nutritional qualities and is even recommended by the WHO for six months after birth [70]. Surprisingly, current studies on AFM1 in human breast milk conducted by Radonić et al. on samples from Serbia revealed alarming contamination levels [71]. Tests conducted on 60 samples showed that around 85% of colostrum and the totality of collected breast milk samples (four-to-eight months after delivery) were found highly contaminated with AFM1 in concentrations beyond tolerable levels [71]. Results of this study and other studies demonstrate the need to raise awareness about AFM1 presence in human milk [72].

## 3. Effective Strategies for AFM1 Reduction

### 3.1. Biological Control and Clay-Based Decontamination Methods

Strategies leading directly or indirectly to AFM1 reduction in milk vary from adopting good agricultural practices to using innovative detoxification methods [51,73,74]. Better management and monitoring of pre- and post-harvest conditions is an essential step to reduce AF contamination [5,75]. Several advanced techniques utilize biological methods such as bacteria, yeasts, and atoxigenic strains to reduce AF contamination in the field and during storage [76,77]. Thus, these “biocontrol” methods lead to the inhibition of fungal growth and AF production [52,78]. In addition, different types of mineral clays have been tested for their capability to bind AF in animal feeds [79,80]. These adsorbents, such as activated carbon (charcoal), zeolite, saponite-rich bentonite, and HSCAS, are able to bind AF, thus reducing AFB1 absorption in the gastrointestinal track and its carry-over as AFM1 in milk [52,81]. For example, the inclusion of HSCAS in dairy feed has resulted in the reduction of up to 50% of the concentration of AFM1 secreted in milk [82]. Furthermore, a recent study conducted by Carraro et al. revealed that bentonite was also effective in reducing AFM1 contamination in milk to levels below the European tolerable limits. Hence, the remaining residual bentonite amounts (0.4%) were in low quantities and showed no harmful effects on human health [83]. There are several limitations of using mineral adsorbents in beverages; they may affect its quality, color, texture, and various other physicochemical properties [54]. Therefore, due to their limitations, several mineral binders are kept for medical usage only [84]. In addition to their effect on food quality, many of these adsorbents are non-specific, non-environmental friendly, and even toxic at high concentration levels [54,85]. All these issues have led researchers to move toward more specific, non-toxic adsorbents, namely microbial ones such as Lactic Acid Bacteria (LAB) and yeasts [86,87,88].

### 3.2. Microbial Decontamination of AFM1

The use of probiotic yeasts and LAB to bind AF in contaminated liquid foods has been widely studied [89,90,91,92,93]. These biological adsorbents may be usually found in different foods including several dairy products such as milk. Their safe status, in addition to the high capability to bind mycotoxins, has lead researchers to test the ability of these adsorbents to bind AFM1 in milk and other liquids [86,90,94]. Consequently, AFM1 binding was reported to be rapid, and the binding percentage varied when changing different factors such as incubation time, temperature, pH, AFM1, and microbial concentrations [38,95]. The use of heat-killed cells is actually more favorable for milk decontamination than viable cells due to the contribution of the latter in product spoilage [86]. In order to assess the binding capability of these adsorbents without interference with the food matrix effect, AFM1 binding assays are initially conducted in buffer solutions such as phosphate-buffered saline (PBS) [74,96,97]. The efficiency of AFM1 binding by microbial adsorbents is detailed in this section.

Table 1, Table 2 and Table 3 represent a summary of the literature using bacteria, yeasts, or a mixture of them for AFM1 decontamination. In addition, the stability of the formed complex in milk or in PBS before (Initial) and after (Final) washing is also highlighted.

#### 3.2.1. Binding Efficiency of Bacterial Strains

AFM1 binding efficiency by different bacterial strains is shown in Table 1. Kabak et al. reported that the binding of AFM1 by viable *Lactobacillus* and *Bifidobacterium* strains in PBS depends on the AFM1 contamination level and incubation time. In addition, they indicated that heating the bacterial pellets did not improve their ability to remove AFM1 from PBS [98]. These findings were not consistent with Bovo et al. (2015), who reported that AFM1 bound by heat-killed *E. avium*, *L. plantarum*, *P. pentosaceus*, *B. lactis*, and *L. gasseri* was significantly greater than the amount bound by viable cells [91]. In fact, these results were in accordance with another study conducted by Assaf et al. in which the percentage of AFM1 bound by *L. rhamnosus* GG in PBS increased significantly, reaching up to 63.08% after heat treatment [74]. This binding increase was explained by the fact that AFM1 may adhere to bacterial cells via electrostatic bonding, thus suggesting that AFM1 is bound to cell wall components, namely polysaccharides and peptidoglycans [99,100]. Furthermore, during heat treatment, the cell wall components are affected by the denaturation of proteins, resulting in an increase in the hydrophobic nature of the cell’s surface in addition to a possible formation of Maillard reaction products [101]. Hence, this denaturation allows AFM1 to bind to bacterial cell wall components that were not accessible when cells were intact [101]. A change in LAB concentration was sufficient to result in a variation of the amount of bound AFM1. According to Kabak et al., a reduction of the bacterial concentration resulted in a significant decrease of bound AFM1 in PBS [102]. This observation was congruent with an Assaf et al. finding, where the binding of AFM1 to *L. rhamnosus* GG greatly increased after increasing the bacterial concentration [74]. Besides, Kabak et al. mentioned that the amount of eliminated AFM1 was not affected by the contamination level of AFM1 in PBS [102].

However, similar findings in milk have been reported by Pierides et al., who demonstrated that heat-killed *L. rhamnosus* GG were able to more efficiently remove AFM1 than viable cells [103]. Accordingly, AFM1 removal in full cream milk (36.6%) was higher compared to skim milk (26.6%), though both were lesser than in PBS. In this regard, the same researchers justified the lower AFM1 removal in milk compared to PBS by the decrease in the availability of free AFM1 possibly associated with casein and other milk contents [103]. In addition, in 2013, Corassin et al. demonstrated that by using a pool of LAB in ultra-high temperature processing (UHT) skim milk, the bound amount of AFM1 has not significantly improved even after an increase in the incubation time [86]. These findings suggested that the binding process of AFM1 with LAB is completed in a fast manner.

#### 3.2.2. Binding Efficiency of Yeast Strains

Upon using different type of yeast strains to bind AFM1 (Table 2), Corassin et al. reported that the binding of AFM1 by *Saccharomyces cerevisiae* in UHT skim milk was significantly higher (up to 92.7%) compared to the binding by LAB pool (up to 11.7%) [86]. Furthermore, in 2018, Abdelmotilib et al. stated that the combination of non-viable yeast strains (*Kluyveromyces lactis* and *S. cerevisiae*) had a higher AFM1 removal effect (85.68%) compared to separate yeast strains (up to 78.74%) [90]. Furthermore, the study showed that the removal of AFM1 by *Kluyveromyces lactis* increased significantly with an increasing yeast concentration [90].

#### 3.2.3. Binding Efficiency of Bacteria and Yeasts Mixed Pools

Upon using a combination of bacterial strains (*L. Plantarum, L. acidophilus*, and *B. bifidum*) and yeast strains (*Kluyveromyces lactis* and *S. cerevisiae*) (Table 3), Abdelmotilib et al. demonstrated that this mixture showed the highest binding of AFM1 in a PBS medium (87.92%) [90]. Similarly Corassin et al. revealed that the amount of AFM1 bound by a mixture of LAB pool and *S. cerevisiae* in UHT skim milk was significantly higher, reaching up to 100% after 60 min of incubation [86]. In fact, this increase in the mixture’s ability to remove AFM1 compared to bacteria or yeast strains was explained by the additive effect of both *S. cerevisiae* and LAB pool, making more accessible sites available for AFM1 fixation. After increasing the total microbial cell concentration in milk by forming a mixed pool of strains, the possible increase in AFM1 retention among microbial cells was not taken into consideration as a potential cause for this binding increase and should be further studied.

#### 3.2.4. AFM1/Microbial Complex Binding Stability

Few studies assessed the stability of the AFM1/microbial complex after successive washing steps (Final binding). In this regard, in 2008, Kabak et al. reported that the binding of AFM1 to bacterial cells was partially reversible, and small amounts of AFM1 (up to 8.54%) were released back into the PBS solution [98]. Furthermore, Bovo et al. revealed that some AFM1 were released back into PBS after several washes but with greater percentages (up to 87%) [91]. Likewise, our observation indicated that after five successive washes, the percentage binding of AFM1 to viable and heat-treated *L. rhamnosus* GG decreased (up to 4.3%) [74]. Several investigators showed that the binding between AFM1 and bacteria is partially reversible, suggesting the implication of non-covalent bonds such as hydrogen bonds and van der Waals interactions [104,105]. A potential clarification of this variation in the released amounts of AFM1 can be explained by the difference in the binding sites between strains or to cross-linked interactions between AFM1 molecules and different microbes [106]. It is noteworthy to mention that the stability of the formed AFM1/microbial complex remains crucial; thus, a stable complex ensures a safe excretion of mycotoxins from the human body [38,107].

### 3.3. Plausible Experimental Variants

Depending on the performed experiments, the AFM1 binding assays using LAB or yeasts have shown some unexplained differences in the percentage of bound AFM1. In this section, we will try to explain the plausible causes of these differences and actions that could be taken in order to better clarify and analyze the given results.

#### 3.3.1. Applied Heat Treatment

The heat treatment of bacteria, yeasts, or mixtures is not performed in a similar way (Table 1, Table 2 and Table 3). Hence, some tests are conducted by heating bacteria at 90–100 °C, while others are conducted by autoclaving at 121 °C. In both terms, the bacteria are heat-killed, but the effect of exerted heat on cell wall components (proteins, peptidoglycan, etc.) and their structures are not taken into consideration. However, depending on heating time, type, and temperature, reversible or irreversible denaturation events may take place [108]. Possibly, a reversible thermal denaturation of proteins or other cell wall components will cause their renaturation after heating [109]. Thus, this may cause an absence of significant changes in AFM1 binding after heat treatment, as shown by Kabak et al. (Table 1). Nevertheless, in another scenario, a variation of the heat rate may cause an irreversible denaturation of proteins and other cell wall components in addition to increasing the number of dissociated electrostatic bonds [74,109,110]. Consequently, it will affect the fixation of AFM1 on its binding sites in the cell wall. These results may explain some of the findings in which an increase in AFM1 binding was observed after heat treatment (Table 1, Table 2 and Table 3). In addition, heating may affect this binding through the formation of Maillard reaction products between proteins, polysaccharides, and peptides [101,111].

#### 3.3.2. Working Temperature

In the conducted experiments for AFM1 binding by microbial adsorbents, the incubation temperature of AFM1-microbial suspension is clearly indicated (Table 1, Table 2 and Table 3). On the other hand, there is a lack of information regarding the working temperature of the carried out experiment that is usually not similar to the fixed incubation temperature of the suspended complex (e.g., 37 °C). Microbial pellets, PBS, and milk may have been stored cold before conducting the binding assay that may take place at room temperature. In addition, for heat-killed bacteria, the temperature of the bacterial pellets may not directly return to room temperature before being suspended in contaminated milk or PBS. Since we are dealing with an electrostatic type of bonding, a variation in the temperature may affect the binding of AFM1 to microbial cells [112]. Thus, for more accuracy, it may be better to indicate the working temperature for the performed assay.

#### 3.3.3. Washing Steps

Testing the stability of the formed AFM1/microbial complex after successive washes was not always considered to be critical, and, for this reason, it was not conducted in all experiments (Table 1, Table 2 and Table 3). Due to the formation of electrostatic bonds, which are weak-to-intermediate in strength (hydrogen and van der Waals), it could be assumed that a certain amount of bound AFM1 may return to suspension (up to 87%) [74,91]. Nevertheless, electrostatic bonding may not be the only reason behind this decrease in bound AFM1 after washing steps. Accordingly, some AFM1 may be retained among bacteria or yeasts even without binding [74]. Therefore, conducting several washes until complete stabilization in the amount of bound AFM1 may be necessary to make appropriate assumptions regarding the actual binding percentage.

#### 3.3.4. Filtration Step

The use of a filtration step to separate microbial adsorbents from AFM1 was not quite favorable due to the retention of some AFM1 that may take place in the filter even without the presence of any adsorbents [74]. Thus, the filtration of a suspension of AFM1 and bacteria may increase the retention of AFM1 in the filter due to membrane pore blockage by the bacteria and the formation of a cake layer. Furthermore, in order to avoid any malfunctions in High Performance Liquid Chromatography (HPLC), some manufacturer’s instruction manuals [113] recommend filtering all samples before AF quantification [114,115,116,117]. In addition, it is worth mentioning that the use of a filtration step for supernatant samples after AFM1 binding may entail errors in the real amount of bound AFM1 [74]. For this reason, it might be better to keep controls for filtration steps at different AFM1 concentrations. In addition, these controls will help in sorting between retained AFM1 in filtration step and unrecovered AFM1 from milk following its clean-up by an immunoaffinity column (IAC). Furthermore, in order to reduce AFM1 retention in the filter, selecting the most appropriate filter membrane (membrane materials, pore size, etc.) may be crucial.

#### 3.3.5. Centrifugation Step

In an AFM1 binding assay, the centrifugation steps are usually conducted to separate bacteria or yeasts from the containing medium (milk, PBS, etc.) [74,103]. For this reason, not much attention was given for the effect of centrifugal speed on AFM1 binding. Therefore, different speeds were used in the conducted experiments (Table 1, Table 2 and Table 3). We highlighted in our previous study that the centrifugation step is implicated in the binding of AFM1 to microbial adsorbents via increasing the contact among them [74]. For this reason, the centrifugation speed and time may affect the amount of bound AFM1, even without changes in other experimental conditions. Hence, the implication of the centrifugation step in AFM1 binding should not be ignored, and further studies may be needed.

#### 3.3.6. Presence of an S-layer

The bacterial S-layer (surface layer) is a layer of thickness between 5 and 25 nm that forms the outermost cell envelope which covers the entire bacteria [118,119]. This layer is composed of proteins or glycoproteins arranged in different shapes in oblique, square, or hexagonal lattice symmetry [120,121]. In addition, the S-layer pore sizes range between 2 and 8 nm in diameter [118,122]. It is possible that this layer acts as a barrier against the entry of AFM1 and binding to the LAB cell wall peptidoglycans or polysaccharides. The potential role of the S-layer in the adsorption or retention of AFM1 should be elucidated after its extraction. It is important to mention that not all LAB have an S-layer [123]. A clear indication of its presence or absence gives a better understanding of the cell wall structure that may affect the binding of AFM1.

#### 3.3.7. Detection and Quantification Techniques

To quantify AFM1 in milk, it is always essential to properly manipulate the samples and choose the appropriate detection method. The reversed-phase HPLC method is a widely used technique for its detection [114,115,116,117]. Other commonly known methods are the enzyme linked immune-sorbent assay (ELISA) and thin-layer chromatography (TLC) [49]. Both the ELISA and TLC methods are cost-effective and easy to handle. For these reasons, they are mainly used as AFM1 screening methods [124]. However, although the use of reverse phase HPLC is more expensive and requires skilled staff, it remains highly accurate with higher sensitivity and specificity [114]. The detection of AFM1 by HPLC is still of great importance due to its high sensitivity, accuracy, reliability, and possibility for column re-usage [114]. In contrast to ELISA, the HPLC method requires a clean-up of AFM1 from milk by using an IAC [116]. Along these lines, various experimental variants may occur at any stage, from sample handling to extraction, detection, or quantification of residual AFM1.

These inaccuracies are not only limited to the binding of AFM1 by microbial adsorbents but may extend to binding of different types of mycotoxins including aflatoxin B1, ochratoxin A, patulin, and other toxins following similar procedures [106,108,125,126].

## 4. Advantages and Limitations

The mechanism of AFM1 decontamination by LAB, yeasts, or mixtures present different advantages over other chemical, physical, or biological methods. This section highlights several advantages of the previously discussed methods in addition to different limitations which may act as a barrier toward their industrial commercialization and that need to be further investigated.

### 4.1. Advantages of Microbial Decontamination

#### 4.1.1. Reduction of AFM1 Bioaccessibility

The use of microbial adsorbents to complex AFM1 may provide an additional strategy to reduce its bioavailability [38]. Hence, a decrease in the amount of free AFM1 for intestinal adsorption will occur. Serrano-Nino et al. revealed that the bioaccessibility of AFM1 in an in vitro digestive model was reduced after AFM1 binding by microbial probiotic strains [127]. Accordingly, *B. bifidum* NRRL B-41410 and *L. acidophilus* NRRL B-4495 were able to reduce the relative bioaccessibility of AFM1 by 45.17% and 32.20%, respectively. Moreover, tests conducted on mice revealed that the concurrent administration of *Lactobacillus plantarum* MON03 (LP) with AFM1 strongly reduced the adverse effects of AFM1 [128]. Therefore, there were no significant differences in tested parameters compared to the control mice [128]. In addition, several studies revealed that the binding of AF to LAB strains increased when simulated in a gastrointestinal environments. As a result of the exposure of LAB cells to bile, an alteration of proteins and phospholipids of the cell envelope may take place, resulting in increased binding [18,89,129,130].

#### 4.1.2. Adsorption Specificity and Effectiveness

The use of either physical methods, such as heating and irradiation [131,132], or chemical methods, including solvent extraction, ammoniation, and ozone treatment [133,134,135,136], for AFM1 removal have many limitations. These detoxification methods are expensive, time consuming, and may cause significant nutritional losses compared to the microbial methods. Thus, microbial decontamination is found to be more effective and highly specific [137]. As observed in Table 1, Table 2 and Table 3, the binding of LAB with AFM1 varied not only between species but also within different strains of the same species, which is an additional indicator of the specificity of this type of binding. Hence, the additional confirmation of AFM1 binding specificity that is supposed to be exerted in specific sites of the microbial cell wall including polysaccharides and peptidoglycan, in addition to its binding mechanisms, needs to be further investigated.

#### 4.1.3. Consumer Product Safety

The use of several probiotics that are “Generally Recognized as Safe” (GRAS) microorganisms for the milk detoxification of AFM1 has made this process safer. It is worth mentioning that probiotics can exert beneficial effects on the host, including consumers of milk and dairy products [138]. The above-discussed methods suggest that supplementing milk with probiotics may be a suitable solution for AFM1 removal in dairy products. In addition, the microbial control of AF production by LAB probiotic strains conferred better protection to milk and other contaminated dairy products during the storage period [52]. Different LAB such as *L. plantarum*, *L. fermentum*, and *L. rhamnosus* are known to be widely used as microbial inoculants for fermentation purposes including milk fermentation [139,140,141]. Therefore, the use of such microbial binders that are commonly found in dairy products and used in their processing is highly preferable. On the other hand, when using chemical or physical agents, some residues may be left in milk that will negatively affect the organoleptic quality of milk, putting the safety of the consumers at risk. Thus, using microbial adsorbents for the elimination of AFM1 from liquids such as milk remains a highly promising strategy.

### 4.2. Limitations of Microbial Decontamination

#### 4.2.1. Microbial Supplementation Limits and Conditions

It is important to indicate that adding microbial agents to milk is acceptable to a certain limit. Current US standards require coliforms no greater than 10 cfu/mL in grade ‘A’ pasteurized fluid milk and a total plate counts of less than 20,000 cfu/mL [142]. Therefore, researchers must be aware of this issue that would be better if taken into consideration when performing the binding assays and fixing the needed microbial concentration. In addition, legislation concerning the total amount of dead and viable bacteria in milk and milk products varies regionally, and respecting these norms is a main concern for the safety of the consumers of dairy products. Adding different amounts of viable LAB, yeasts, or a mixture of both to milk is not quite favorable due to their uncontrolled proliferation. For example, yeasts such as *Kluyveromyces* sp. and *Saccharomyces* sp. usually cause milk spoilage by fermenting milk lactose [143].

#### 4.2.2. Removal of Supplemented Microorganisms

If the concentrations of microbial agents necessary for AFM1 decontamination surpass the allowed limits, then they cannot remain in milk and a treatment for their removal is required. The removal of LAB or yeasts that were previously supplemented into milk using a filtration step is not easy to achieve due to several limiting factors. Within the required milk treatment for microbial removal, low membrane selectivity may take place in addition to its high operating costs [144]. Furthermore, size similarities between different milk components such as microbial adsorbents and fat can make this process harder to accomplish at low cost, and additional steps may be required. Thus, a proportion of the native milk fat globules which are similar in size to bacteria must be removed by centrifugal separation before conducting a microfiltration step [144]. It appears that this removal process is expensive, and important milk components may be lost, which means that they have to be re-supplemented later due to their significance to consumers.

#### 4.2.3. Binding Reversibility

As previously reported in this review, the binding of AFM1 to microbial adsorbents is partially reversible. Hence, the non-covalent type of binding between microbial binders and AFM1 may be a main concern related to its industrial application. Since milk contamination by AFM1 and its maximum tolerable limits are not similar worldwide, the amount of supplemented yeasts, bacteria, or mixture of both in AFM1-contaminated milk needs to be regularly assessed. Thus, estimating the amounts of needed adsorbents is hard to achieve due to the partial reversibility of this type of bonding. Additionally, the stability of AF binding in milk or other liquids may differ according to different environmental condition including storage time, pH, milk temperature, and concentration of the used microbial adsorbents [145]. It remains tough to continuously monitor the amounts of free AFM1 and estimate the concentration of microbial agents in an unstable environment, especially when microbial additives bound to AFM1 are destined to be retained in milk.

## 5. Prospective Industrial Applications

Numerous microbial adsorbents have been tested in order to determine their potential ability to bind AF in milk and other dairy products, but, so far, researchers have not been able to commercially implement a fully reliable method. For this reason, several prospective methods for industrial applications are discussed in this section.

### 5.1. Microbial Fixation on Support or Membrane

The proposed method reported by Foroughi et al. in 2018 consists of immobilizing yeast such as *Saccharomyces cerevisiae* on perlite support to detoxify AFM1-contaminated milk [146]. The results showed a significant reduction in AFM1 concentration for all tested milk samples with various initial AFM1 contents. The highest reduction of AFM1 obtained was 81.3% after 80 min of milk circulation in the biofilter. This study revealed the high capability of immobilized yeast cells to detoxify AFM1 without any changes of its physicochemical properties. These promising results may be used for additional research such as fixing effective quantities of LAB, yeasts, or mixtures on a support or membrane that may be used to detoxify AFM1 by passing contaminated liquids through or over it. The formation of customized biofilters or cartridges containing these biological adsorbents may be more suitable for industrial application. Therefore, microbial cell immobilization is a remarkable method that may lead to different practical applications addressing not only AFM1 contamination in dairy products but also mycotoxins decontamination in beverages.

### 5.2. Microbial Biofilm Formation

A potential solution to the retention of microbial agents used for AFM1 decontamination in milk was shown in a study conducted by Assaf et al. in 2019 [50]. Tests were carried out to examine the ability of biofilm formed by probiotic LAB strains in tubes or in plates to eliminate AFM1. Hence, *L. rhamnosus* GG biofilm was able to significantly remove (up to 60.74%) AFM1 from contaminated whole milk. In addition, no significant difference in milk protein content was observed after AFM1 binding. Therefore, passing contaminated milk through or over the resultant biofilm for AFM1 decontamination could be a direct application of this method. It is important to mention that probiotic LAB and yeasts are able to form biofilms on different type of surfaces [147]. As such, their emerging applications in mycotoxin decontamination should be further elucidated.

### 5.3. Customized Rotating Mixer

As previously shown, an increase of AFM1 exposure to microbial adsorbents may affect the amount of AFM1 bound by LAB [74]. Thus, the binding of AFM1 to heat-killed *L. rhamnosus* GG increased when coupled to a mixing step such as pipetting [74]. For this reason, it may be suitable to use a customized rotating mixer that can increase the contact between AFM1 and microbial binders, thus decreasing the amount of microbial inoculum needed and the decontamination time. This procedure may be coupled with a filtration step to remove the supplemented microbial agents. Noting that even without any microbial supplementation, the increase of contact between AFM1 and milk components including LAB and proteins such as casein may result in an increase in the binding of free AFM1, thereby decreasing AFM1 bioavailability in contaminated milk [148,149,150].

## 6. Conclusions

Among all mycotoxins, the group of aflatoxins has received much attention due to their severe impact on human and animal health. In fact, numerous studies have investigated various microbial agents for their potential to bind AFM1. In this review, we aimed to investigate AFM1 decontamination methods by using microbial adsorbents and to emphasize the role of different experimental variants on complex binding and stability. Accordingly, this work highlights several experimental parameters that should be taken into consideration to optimize the binding of AFM1 in milk and other liquids. The decontamination of AFM1 using microbial adsorbents is still under vigorous investigation, and a better understanding of its binding mechanism and stability is needed. In addition, considerable testing of the physiochemical properties of milk after decontamination needs to be elucidated. Further studies on using these agents for AFM1 decontamination are still needed before the industrial implementation of the developed methods on milk products. This review highlights the use of different AFM1 decontamination methods and their plausible inaccuracies, thus answering some essential questions for a better understanding and improvement of these methods.

## Figures and Tables

**Table 1:** Summary of studies evaluating AFM1 binding by different bacterial strains.

Type	Strain	[AFM1]—[Cells]	Solution	Treatment	Incubation Time (37 °C)	Centrifugal Force (g/rpm)	Initial Binding (%)	Final Binding (%)	Reference
**Bacteria**	*L. rhamnosus GG*	[50 μg/L]—[10^10^]	PBS	Viable	18 h	10 min—3000 g	55.62 ± 0.2 aλ	51.32 ± 0.3 a’*	[59]
	*L. rhamnosus GG*	[50 μg/L]—[10^10^]	PBS	90 °C—1 h	18 h	10 min—3000 g	63.08 ± 0.3 a	59.67 ± 0.4 a’*	
	*L. rhamnosus GG*	[100 μg/L]—[5 × 10^8^]	PBS	Viable	18 h	10 min—3000 g	1.38 ± 0.2 λ	0.51 ± 0.23*	
	*L. acidophilus NCC 36*	[5 μg/L]—[10^7^]	PBS	Viable	0 h	15 min—3000 g	3.44 ± 3.04 β	-	[77]
	*L. acidophilus NCC 36*	[5 μg/L]—[10^8^]	PBS	Viable	0 h	15 min—3000 g	22.23 ± 10.76 β	-	
	*L. acidophilus NCC 36*	[5 μg/L]—[10^8^]	PBS	Viable	24 h	15 min—3000 g	22.24 ± 4.67	-	
	*L. acidophilus NCC 36*	[20 μg/L]—[10^8^]	PBS	Viable	0 h	15 min—3000 g	24.78 ± 1.39	-	
	*L. acidophilus NCC 36*	[20 μg/L]—[10^8^]	PBS	Viable	24 h	15 min—3000 g	23.10 ± 5.19	-	
	*L. acidophilus NCC 36*	[5 μg/L]—[10^8^]	PBS	90 °C—50 min	0 h	15 min—3000 g	26.38 ± 4.99	-	
	*L. acidophilus NCC 36*	[5 μg/L]—[10^8^]	PBS	90 °C—50 min	24 h	15 min—3000 g	25.29 ± 5.03	-	
	*L. acidophilus NCC 36*	[20 μg/L]—[10^8^]	PBS	90 °C—50 min	0 h	15 min—3000 g	26.22 ± 4.93	-	
	*L. acidophilus NCC 36*	[20 μg/L]—[10^8^]	PBS	90 °C—50 min	24 h	15 min—3000 g	24.50 ± 4.40	-	
	*L. acidophilus NCC 36*	[5 μg/L]—[10^8^]	Reconstituted skim milk	Heat-killed	4 h	15 min—1800 g	23.73 ± 2.52	-	
	*L. acidophilus NCC 36*	[10 μg/L]—[10^8^]	Reconstituted skim milk	Heat-killed	4 h	15 min—1800 g	24.13 ± 4.67	-	
	*L. acidophilus NCC 36*	[20 μg/L]—[10^8^]	Reconstituted skim milk	Heat-killed	4 h	15 min—1800 g	25.07 ± 7.96	-	
	*L. acidophilus NCC 36*	[5 μg/L]—[10^8^]	Reconstituted skim milk	Viable	4 h	15 min—1800 g	22.70 ± 3.36	-	
	*L. rhamnosus*	[5 μg/L]—[10^8^]	PBS	Viable	0 h	15 min—3000 g	20.21 ± 6.16	-	
	*L. rhamnosus*	[5 μg/L]—[10^8^]	PBS	Viable	24 h	15 min—3000 g	22.16 ± 7.14	-	
	*L. rhamnosus*	[20 μg/L]—[10^8^]	PBS	Viable	0 h	15 min—3000 g	22.88 ± 7.11	-	
	*L. rhamnosus*	[20 μg/L]—[10^8^]	PBS	Viable	24 h	15 min—3000 g	21.64 ± 1.66	-	
	*L. rhamnosus*	[5 μg/L]—[10^8^]	PBS	90 °C—50 min	0 h	15 min—3000 g	23.37 ± 4.81	-	
	*L. rhamnosus*	[5 μg/L]—[10^8^]	PBS	90 °C—50 min	24 h	15 min—3000 g	24.16 ± 3.33	-	
	*L. rhamnosus*	[20 μg/L]—[10^8^]	PBS	90 °C—50 min	0 h	15 min—3000 g	27.78 ± 7.50	-	
	*L. rhamnosus*	[20 μg/L]—[10^8^]	PBS	90 °C—50 min	24 h	15 min—3000 g	26.69 ± 5.48	-	
	*L. rhamnosus*	[5 μg/L]—[10^8^]	Reconstituted skim milk	Heat-killed	4 h	15 min—1800 g	25.13 ± 6.19	-	
**Bacteria**	*L. rhamnosus*	[10 μg/L]—[10^8^]	Reconstituted skim milk	Heat-killed	4 h	15 min—1800 g	22.86 ± 9.33	-	[77]
	*L. rhamnosus*	[10 μg/L]—[10^8^]	Reconstituted skim milk	Heat-killed	4 h	15 min—1800 g	22.86 ± 9.33	-	
	*L. rhamnosus*	[20 μg/L]—[10^8^]	Reconstituted skim milk	Heat-killed	4 h	15 min—1800 g	26.27 ± 1.92	-	
	*L. rhamnosus*	[5 μg/L]—[10^8^]	Reconstituted skim milk	viable	4 h	15 min—1800 g	21.74 ± 3.56	-	
	*B. bifidum Bb13*	[5 μg/L]—[10^8^]	PBS	Viable	0 h	15 min—3000 g	23.48 ± 6.12	-	
	*B. bifidum Bb13*	[5 μg/L]—[10^8^]	PBS	Viable	24 h	15 min—3000 g	26.65 ± 2.60	-	
	*B. bifidum Bb13*	[20 μg/L]—[10^8^]	PBS	Viable	0 h	15 min—3000 g	24.77 ± 4.35	-	
	*B. bifidum Bb13*	[20 μg/L]—[10^8^]	PBS	Viable	24 h	15 min—3000 g	26.33 ± 1.82	-	
	*B. bifidum Bb13*	[5 μg/L]—[10^8^]	PBS	90 °C—50 min	0 h	15 min—3000 g	27.74 ± 2.97	-	
	*B. bifidum Bb13*	[5 μg/L]—[10^8^]	PBS	90 °C—50 min	24 h	15 min—3000 g	25.12 ± 5.33	-	
	*B. bifidum Bb13*	[20 μg/L]—[10^8^]	PBS	90 °C—50 min	0 h	15 min—3000 g	28.97 ± 3.49	-	
	*B. bifidum Bb13*	[20 μg/L]—[10^8^]	PBS	90 °C—50 min	24 h	15 min—3000 g	27.31 ± 1.82	-	
	*B. bifidum Bb13*	[5 μg/L]—[10^8^]	Reconstituted skim milk	Heat-killed	4 h	15 min—1800 g	25.41 ± 4.60	-	
	*B. bifidum Bb13*	[10 μg/L]—[10^8^]	Reconstituted skim milk	Heat-killed	4 h	15 min—1800 g	25.64 ± 3.18	-	
	*B. bifidum Bb13*	[20 μg/L]—[10^8^]	Reconstituted skim milk	Heat-killed	4 h	15 min—1800 g	27.31 ± 1.82	-	
	*L. plantarum*	[150 μg/L]—[10^10^]	PBS	Viable	15 min	15 min—1800 g	5.60 ± 0.45 bA	3.71 ± 0.02 b’*	[69]
	*L. plantarum*	[150 μg/L]—[10^10^]	PBS	100 °C—1 h	15 min	15 min—1800 g	13.11 ± 0.89 b	8.229 ± 0.03 b’*	
	*L. plantarum*	[150 μg/L]—[10^10^]	PBS	viable	24 h	15 min—1800 g	8.09 ± 1.33 cA	5.571 ± 0.06 c’*	
	*L. plantarum*	[150 μg/L]—[10^10^]	PBS	100 °C—1 h	24 h	15 min—1800 g	14.14 ± 1.03 c	7.60 ± 0.03 c’*	
	*E. avium*	[150 μg/L]—[10^10^]	PBS	Viable	15 min	15 min—1800 g	7.36 ± 1.10 d	5.19 ± 0.08 d’*	
	*E. avium*	[150 μg/L]—[10^10^]	PBS	100 °C—1 h	15 min	15 min—1800 g	12.42 ± 2.20 d	7.070 ± 0.126 d’*	
	*E. avium*	[150 μg/L]—[10^10^]	PBS	viable	24 h	15 min—1800 g	6.64 ± 1.40 e	2.69 ± 0.06 e’*	
	*E. avium*	[150 μg/L]—[10^10^]	PBS	100 °C—1 h	24 h	15 min—1800 g	13.13 ± 2.14 e	7.446 ± 0.13 e’*	
**Bacteria**	*P. pentosaceus*	[150 μg/L]—[10^10^]	PBS	Viable	15 min	15 min—1800 g	8.68 ± 1.24 f	5.36 ± 0.07 f’*	[69]
	*P. pentosaceus*	[150 μg/L]—[10^10^]	PBS	100 °C—1 h	15 min	15 min—1800 g	15.16 ± 2.40 f	8.65 ± 0.14 f’*	
	*P. pentosaceus*	[150 μg/L]—[10^10^]	PBS	viable	24 h	15 min—1800 g	7.76 ± 0.95 g	5.45 ± 0.079 g’*	
	*P. pentosaceus*	[150 μg/L]—[10^10^]	PBS	100 °C—1 h	24 h	15 min—1800 g	13.86 ± 1.01 g	7.86 ± 0.07 g’*	
	*L. gasseri*	[150 μg/L]—[10^10^]	PBS	Viable	15 min	15 min—1800 g	21.37 ± 2.76 h	16.91 ± 0.117 h’*	
	*L. gasseri*	[150 μg/L]—[10^10^]	PBS	100 °C—1 h	15 min	15 min—1800 g	32.57 ± 1.96 h	20.6 ± 0.07 h’*	
	*L. gasseri*	[150 μg/L]—[10^10^]	PBS	Viable	24 h	15 min—1800 g	22.77 ± 1.81 i	14.51 ± 0.017 i’*	
	*L. gasseri*	[150 μg/L]—[10^10^]	PBS	100 °C—1 h	24 h	15 min—1800 g	32.30 ± 0.98 i	20.77 ± 0.012 i’*	
	*L. bulgaricus*	[150 μg/L]—[10^10^]	PBS	Viable	15 min	15 min—1800 g	30.22 ± 1.43 kB	19.05 ± 0.05 k’*	
	*L. bulgaricus*	[150 μg/L]—[10^10^]	PBS	100 °C—1 h	15 min	15 min—1800 g	36.32 ± 1.09 k	23.81 ± 0.05 k’*	
	*L. bulgaricus*	[150 μg/L]—[10^10^]	PBS	Viable	24 h	15 min—1800 g	33.54 ± 1.56 B	18.02 ± 0.10 p’*	
	*L. bulgaricus*	[150 μg/L]—[10^10^]	PBS	100 °C—1 h	24 h	15 min—1800 g	33.93 ± 1.91	23.5 ± 0.08 p’*	
	*L. rhamnosus*	[150 μg/L]—[10^10^]	PBS	Viable	15 min	15 min—1800 g	17.13 ± 3.01 lC	14.96 ± 0.05 l’*	
	*L. rhamnosus*	[150 μg/L]—[10^10^]	PBS	100 °C—1 h	15 min	15 min—1800 g	35.69 ± 3.13 l	23.02 ± 0.13 l’*	
	*L. rhamnosus*	[150 μg/L]—[10^10^]	PBS	Viable	24 h	15 min—1800 g	27.79 ± 2.67 mC	16.51 ± 0.05 m’*	
	*L. rhamnosus*	[150 μg/L]—[10^10^]	PBS	100 °C—1 h	24 h	15 min—1800 g	45.67 ± 1.65 m	22.45 ± 0.063 m’*	
	*B. lactis*	[150 μg/L]—[10^10^]	PBS	Viable	15 min	15 min—1800 g	16.89 ± 2.01 n	13.34 ± 0.115 n’*	
	*B. lactis*	[150 μg/L]—[10^10^]	PBS	100 °C—1 h	15 min	15 min—1800 g	36.56 ± 2.46 n	23 ± 0.22 n’*	
	*B. lactis*	[150 μg/L]—[10^10^]	PBS	Viable	24 h	15 min—1800 g	23.62 ± 4.13 o	13.71 ± 0.29 o’*	
	*B. lactis*	[150 μg/L]—[10^10^]	PBS	100 °C—1 h	24 h	15 min—1800 g	35.84 ± 3.85 o	21.22 ± 0.316 o’*	
	*L. rhamnosus strain GG*	[150 μg/L]—[10^10^]	skim milk	Viable	≈16 h	15 min—3500g	18.8 ± 1.9 pD	-	[78]
	*L. rhamnosus strain GG*	[150 μg/L]—[10^10^]	skim milk	100 °C—1 h	≈16 h	15 min—3500 g	26.6 ± 3.2 pE	-	
	*L. rhamnosus strain GG*	[150 μg/L]—[10^10^]	full cream milk	Viable	≈16 h	15 min—3500 g	26.0 ± 1.5 qD	-	
	*L. rhamnosus strain GG*	[150 μg/L]—[10^10^]	full cream milk	100 °C—1 h	≈16 h	15 min—3500 g	36.6 ± 1.1 qE	-	
	LAB pool (*L.delbrueckii spp. Bulgaricus, L. rhamnosus and B.lactis*)	[0.5 μg/L]—[10^10^]	UHT skim milk	100 °C—1 h	30 min	15 min—1800 g	11.5 ± 2.3	-	[66]
	LAB pool (*L.delbrueckii* spp. *Bulgaricus, L. rhamnosus* and *B.lactis*)	[0.5 μg/L]—[10^10^]	UHT skim milk	100 °C—1 h	60 min	15 min—1800 g	11.7 ± 4.4	-	[66]

Results are the average ± SD for triplicates sample. Two-way ANOVA was conducted. Indicates a significant binding differences (*p* < 0.05) between: (*) Initial and final binding % of viable or heat-killed bacteria. (a, b, c, d, e, f, g, h, I, j, k, l, m, n, o, p, q, r, s, t, q) viable and heat-killed bacteria before washing. (a’, b’, c’, d’, e’, f’, g’, h’, i’, j’, k’, l’, m’, n’, o’) viable and heat-killed bacteria after washing. (A, B, C, D, E) bacteria treated at different incubation time. (λ, β) bacteria treated at different AFM1 concentration.

**Table 2:** Summary of studies evaluating the AFM1 binding by different yeast strains.

Type	Strain	[AFM1]—[Cells]	Solution	Treatment	Incubation Time (37 °C)	Centrifugal Force (g/rpm)	Initial Binding (%)	Reference
**Yeast**	*S. cerevisiae*	[0.5 μg/L]—[10^9^]	UHT skim milk	100 °C—1 h	30 min	15 min—1800 g	90.3 ± 0.3 A	[66]
	*S. cerevisiae*	[0.5 μg/L]—[10^9^]	UHT skim milk	100 °C—1 h	60 min	15 min—1800 g	92.7 ± 0.7 A	
	*Kluyveromyces lactis*	[50 μg/L]—[10^9^]	PBS	121 °C—10 min	72 h	15 min—6000 rpm	60.14 ± 2.5 λ	[68]
	*Kluyveromyces lactis*	[50 μg/L]—[5 × 10^9^]	PBS	121 °C—10 min	72 h	15 min—6000 rpm	69.14 ± 1.8 λ	
	*S. cerevisiae*	[50 μg/L]—[10^9^]	PBS	121 °C—10 min	72 h	15 min—6000 rpm	64.52 ± 1.83 β	
	*S. cerevisiae*	[50 μg/L]—[5 × 10^9^]	PBS	121 °C—10 min	72 h	15 min—6000 rpm	78.74 ± 1.82 β	
	CYS-NV (*S. cerevisiae* + *k. lactis*)	[50 μg/L]—[5 × 10^9^]	PBS	121 °C—10 min	72 h	15 min—6000 rpm	85.68 ± 1.84	

Results are the average ± SD for triplicates sample. Two-way ANOVA was conducted. Indicates a significant binding differences (*p* < 0.05) between: (A) Yeast treated at different incubation time. (λ, β) yeast treated at different AFM1 concentration.

**Table 3:** Summary of studies evaluating AFM1 binding by a mixture of yeasts and bacterial strains.

Type	Strain	[AFM1]—[Cells]	Solution	Treatment	Incubation Time (37 °C)	Centrifugal Force (g/rpm)	Initial Binding (%)	Reference
**Mixture**	LAB pool + *S. cerevisiae*	[0.5 μg/L]—[10^10^] LAB pool + [10^9^] *S. cerevisiae*	UHT skim milk	100 °C—1 h	30 min	15 min 1800 g	91.7 ± 0.5 A	[66]
	LAB pool + *S. cerevisiae*	[0.5 μg/L]—[10^10^] LAB pool + [10^9^] *S. cerevisiae*	UHT skim milk	100 °C—1 h	60 min	15 min 1800 g	100.0 ± 0.0 A	
	CPYS-NV (*B. bifidum* + *L. acidophilus* + *L. Plantarum* + *S. cerevisiae* + *k. lactis*)	[50 μg/L]—[5 × 10^9^]	PBS	121 °C—10 min (b)—20 min (y)	72 h	-	87.92 ± 1.10	[68]
	CPYS-NV (*B. bifidum* + *L. acidophilus* + *L. Plantarum* + *S. cerevisiae* + *k. lactis*)	[50 μg/L]—[5 × 10^9^]	skim milk	121 °C—10 min (b) 121 °C—20 min (y)	12 h	-	80.56 ± 2.19 B	
		[50 μg/L]—[5 × 10^9^]	skim milk	121 °C—10 min (b) 121 °C—20 min (y)	24 h	-	86.64 ± 1.5 B	
		[50 μg/L]—[5 × 10^9^]	skim milk	121 °C—10 min (b) 121 °C—20 min (y)	48 h	-	88.6 ± 1.3 C	
		[50 μg/L]—[5 × 10^9^]	skim milk	121 °C—10 min (b) 121 °C—20 min (y)	72 h	-	90.88 ± 1.09 C	

Results are the average ± SD for triplicates sample. Two-way ANOVA was conducted. Indicates a significant binding differences (*p* < 0.05) between: (A, B, C) cells treated at different incubation time. (b): Bacterial strains (y): Yeast strains.
